# Diagnosis Value of the Serum Amyloid A Test in Neonatal Sepsis: A Meta-Analysis

**DOI:** 10.1155/2013/520294

**Published:** 2013-08-05

**Authors:** Haining Yuan, Jie Huang, Bokun Lv, Wenying Yan, Guang Hu, Jian Wang, Bairong Shen

**Affiliations:** ^1^Center for Systems Biology, Soochow University, Suzhou 215006, China; ^2^Affiliated Children's Hospital of Soochow University, Suzhou 225121, China; ^3^Suzhou Zhengxing Translational Biomedical Informatics Ltd., Taicang 215400, China

## Abstract

Neonatal sepsis (NS), a common disorder for humans, is recognized as a leading global public health challenge. This meta-analysis was performed to assess the accuracy of the serum amyloid A (SAA) test for diagnosing NS. The studies that evaluated the SAA test as a diagnotic marker were searched in Pubmed, EMBASE, the Cochrane Library, and Google Network between January 1996 and June 2013. A total of nine studies including 823 neonates were included in our meta-analysis. Quality of each study was evaluated by the quality assessment of diagnostic accuracy studies tool (QUADAS). The SAA test showed moderate accuracy in the diagnosis of NS both at the first suspicion of sepsis and 8–96 h after the sepsis onset, both with Q* = 0.91, which is similar to the PCT and CRP tests for the diagnosis of NS in the same period. Heterogeneity between studies was also explained by cut-off point, SAA assay, and age of included neonates. On the basis of our meta-analysis, therefore, SAA could be promising and meaningful in the diagnosis of NS.

## 1. Introduction

Neonatal sepsis (NS) is recognized as a leading global public health challenge because of the important contribution to neonatal morbidity and mortality in both low and high income countries [1–3]. It is mainly responsible for most of neonatal deaths, and the incidence of NS is about 3–40 per 1000 live births, the mortality rate of whom varies from 9% to 20% of affected neonates [4, 5]. 

Neonatal sepsis may be defined, both clinically and/or microbiologically, by positive blood and/or cerebrospinal fluid cultures. Early gradual signs and symptoms of NS are often indefinite and subtle, especially at the onset, which is easily confused with other common noninfectious causes [6]. Clinicians often treat with a broad-spectrum antibiotic and prolong treatment with empirical antibiotics, which are associated with adverse outcomes. Furthermore, physical signs are used to identify neonates at risk of sepsis with limited specificity, generally resulting in large number of unnecessary referrals, and antibiotics treatment in the setting of negative cultures may not be benign [7, 8]. Early diagnosis and treatment are vital to improve gradual outcomes; if diagnosed early and with aggressive supportive care, it is possible to save most cases of NS [9]. To this aim, clinicians have sought reliable markers to detect early NS for a long time and to exclude diseases of noninfectious origin [10, 11]. Till now, a large number of markers have been proposed for early diagnosis of sepsis [12], especially C-reactive protein (CRP) and procalcitonin (PCT). CRP is an acute-phase protein found in the blood that is produced by the liver because of infection or tissue injury, while PCT is a 116-amino acid peptide involved as a precursor in calcium homeostasis, and both of them have been widely used as useful markers for the diagnosis of neonatal sepsis [13–17]. However, the specificity and the value of CRP and PCT still have challenges; thus, there is a continuous need for searching for better biomarkers of sepsis.

Serum amyloid A (SAA), the precursor protein in inflammation-associated reactive amyloidosis, whose level in the blood increases up to 1000 fold in response to information, is synthesized in the liver. SAA is also an acute phase reactant like PCT and CRP, which has been proven to be a prognostic marker in late-onset sepsis in preterm infants [18–20]. Arnon et al. [21] reported that SAA had an overall better diagnostic accuracy than CRP for predicting early onset sepsis. Also, they showed that SAA was a useful inflammatory marker during late-onset sepsis in preterm infants [22]. However, some studies showed an opposite opinion [23, 24]. In view of this contradiction, a more comprehensive study is needed to discuss the accuracy of the SAA test for the diagnosis of NS. 

Our primary objective is to systematically and quantitatively evaluate all published studies about the diagnostic use of SAA for NS.

## 2. Methods

### 2.1. Retrieval and Selection of Studies

The common approach of computer-aided literature search is used to search PUBMED, EMBASE (http://www.embase.com/), the Cochrane Library (http://www.thecochranelibrary.com/view/0/index.html), and Google Network for relevant citations from January 1996 to June 2013. Our search terms included “serum amyloid A,” “SAA,” “sepsis,” “septicemia,” “neonate,” “newborn,” “infant,” and mutual combinations. We have examined the references of known articles to fully retrieve.

The following criteria were applied to identify studies for inclusion in our meta-analysis: (1) studies that assessed the diagnostic accuracy of the SAA test on NS; (2) studies providing both sensitivity and specificity or sufficient information to construct the 2 × 2 contingency tables; and (3) studies with the sample containing only neonates. Articles including studies that evaluated SAA levels as diagnostic markers for NS are appropriate. The gold standard for the diagnosis of neonatal sepsis is microbial culture blood or other sterile body fluids in these included studies. Furthermore, the change of the SAA in the research sample is the index test for the diagnosis of neonatal sepsis. Selection of articles was performed by two investigators independently to ensure the high accuracy.

### 2.2. Data Extraction

We extracted data from selected articles, which include first authors, years of publication, study population, region, methods of SAA assay, diagnostic cut-off point and time, and methods quality. Accurate data was extracted to construct 2 × 2 table at a specific time.

### 2.3. Quality Assessment

Quality assessment of studies was performed based on the quality assessment of diagnostic accuracy studies (QUADAS) tool [25]. This tool consists of 11 key items. Each item was assessed by scoring it as “low,” “high,” “unclear,” which are phrased to the answer, such as “yes” indicates low risk of bias.

### 2.4. Statistical Analysis

The statistical analysis was performed using Review Manager 5.0 and Meta-DiSc 1.4 software. Studies included in the meta-analysis were divided into two groups according to the time of SAA test for diagnosis of NS. The neonates at the onset of sepsis were set as a group, and those 8–96 h after onset were set as another. The sensitivity, specificity, and diagnostic odds ratio (OR) with corresponding 95% confidence intervals (CI) were calculated for each study. Meanwhile, the pooled sensitivity, specificity and diagnosis OR were also calculated for each group. The diagnostic OR expresses how much greater are the odds of having sepsis for neonates with a positive test result than for neonates with a negative test result [26]. Heterogeneity among included studies is assessed by using the Cochrane *Q* statistic and quantified with the *I* ^2^ lying between 0% and 100% [27]. In general, *I* ^2^ (>50%) shows that heterogeneity among studies produce some impact, whereas *I* ^2^ (<50%) shows that homogeneity is good for the reliability of meta-analysis. We further performed sensitivity analysis to explore the reasons of heterogeneity and examined characteristics of included studies. To summarize these results, we constructed a summary receiver operator characteristic (SROC) curve, which shows the relationship between sensitivity and the proportion of false positives (1-specificity). Q* values, defined by the point where sensitivity equals specificity, were calculated from the SROC curves. Meanwhile, the area under SROC curve (AUC) was also calculated to show the probability of the correctly ranked diagnostic test values for a random pair of diseased and nondiseased subjects [28].

## 3. Results

### 3.1. Characteristics and Quality of the Included Studies

**Figure 1:**
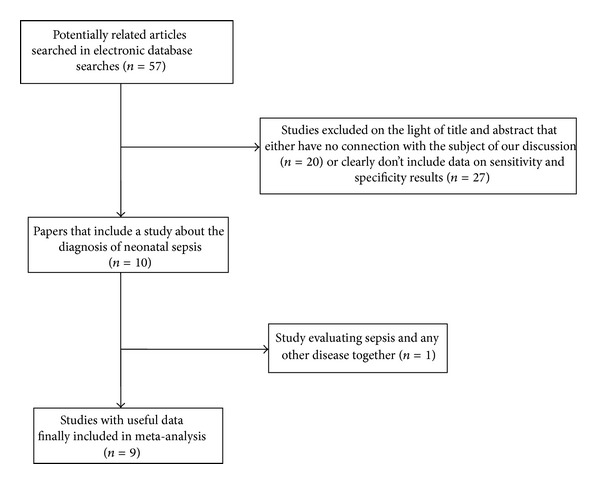
Flow chart of study evaluation and inclusion in the meta-analysis of studies involving diagnosis of neonatal sepsis using a SAA test.

The literature search was performed as described, and 57 potentially relevant articles were identified. Only 9 articles met our inclusion criteria.  Figure 1 shows the process of selecting studies. Detailed characteristics and data of each included study are presented in Tables 1 and 2, respectively.

**Figure 2:**
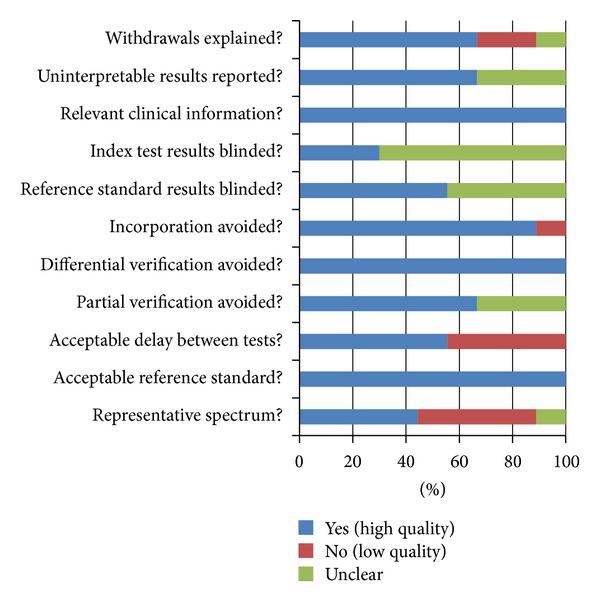
Summary of the methodological quality assessment of the included studies according to 11 QUADAS tool criteria, which are presented as percentages.

All the conditions and methods of the included studies, as shown in Figure 2, were used for different quality assessment of diagnostic accuracy. Each included study was strictly judged on the basis of 11 QUADAS tool criteria [25]: withdrawals explained; uninterpretable results reported; relevant clinical information; index test results blinded; reference standard results blinded; incorporation avoided; differential verification avoided; partial verification avoided; Acceptable delay between tests; acceptable reference standard; and representative spectrum. We also appraise them quantitatively according to 11 QUADAS tool criteria, as shown in Table 2.

### 3.2. Accuracy of the SAA Test in the Diagnosis of NS

 Nine articles, which meet inclusion criteria, estimate the use of the SAA test in the diagnosis of NS. We set studies at the first suspicion of NS as a group and those at 8–96 h after sepsis onset as another group on the basis of time point for the SAA test.

**Figure 3:**
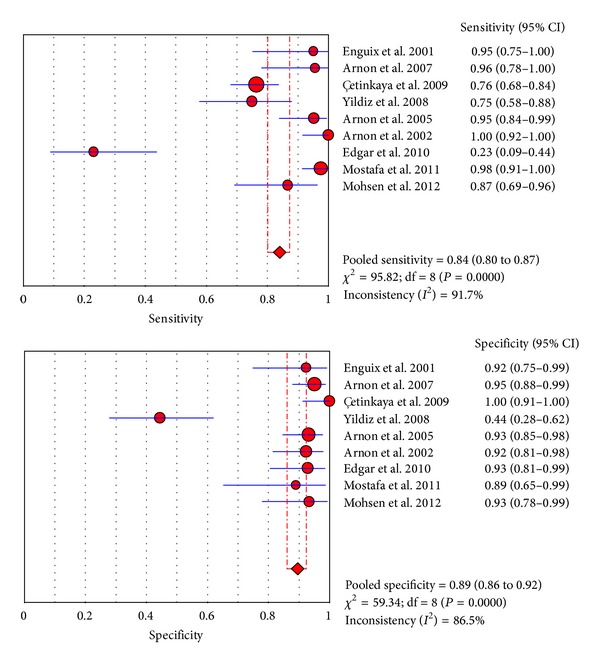
Forest plot [46] for sensitivity and specificity of the SAA test to diagnose neonatal sepsis at the first suspicion of sepsis.

Nine studies from included papers evaluated the use of the SAA test at the first suspicion of sepsis. The sensitivity ranged from 23% to 100% (pooled sensitivity: 0.84, 95% CI 80%–87%), whereas specificity ranged from 44% to 100% (pooled sensitivity: 0.89, 95% CI 86%–92%). The detailed descriptions are shown in Figure 3. We found significant heterogeneity among studies (sensitivity, *I* ^2^ = 92.7%; specificity, *I* ^2^ = 86.5%), which indicated that patient selection or other covariates might be responsible for heterogeneity. 

**Figure 4:**
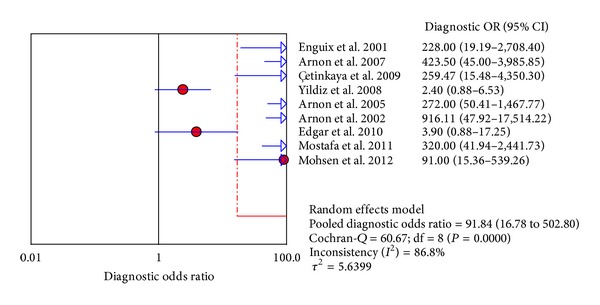
Forest plot for diagnostic OR of the SAA test to diagnose neonatal sepsis at the first suspicion of sepsis.

**Figure 5:**
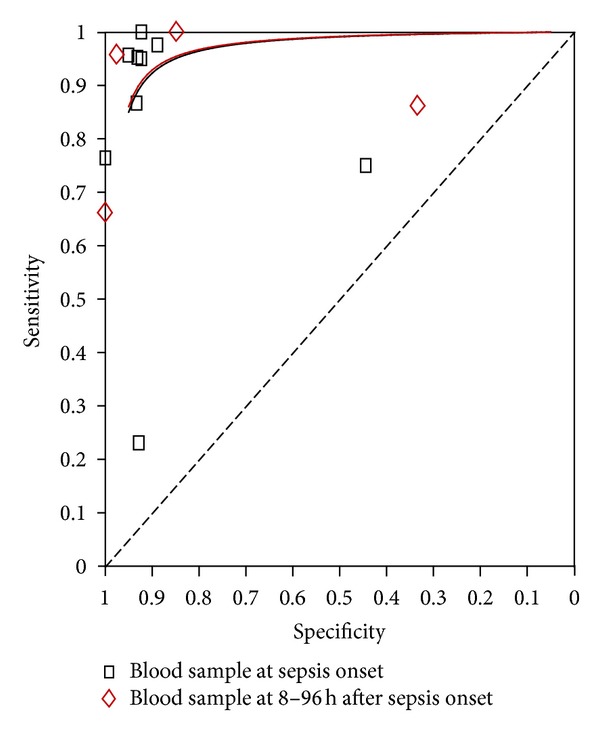
Summary receiver operating characteristic (SROC) curve of the SAA test for the diagnosis of neonatal sepsis.

The value of DOR of SAA was 91.84 (95% CI, 16.78–502.80), as shown in the forest plot of Figure 4. Among these studies, we also detected significant heterogeneity (*I* ^2^ = 86.8%). The corresponding SROC curve was plotted in Figure 5, which shows the AUC was 0.96 with standard error of 0.02, and the pooled diagnostic accuracy (Q*) was 0.90 with standard error of 0.03, showing a high overall accuracy of SAA for NS.

**Figure 6:**
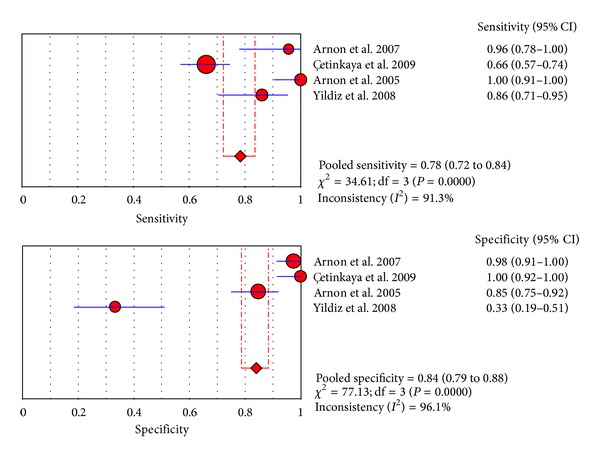
Forest plots for sensitivity and specificity of the SAA test to diagnose neonatal sepsis at 8–96 h after the first suspicionof sepsis.

**Figure 7:**
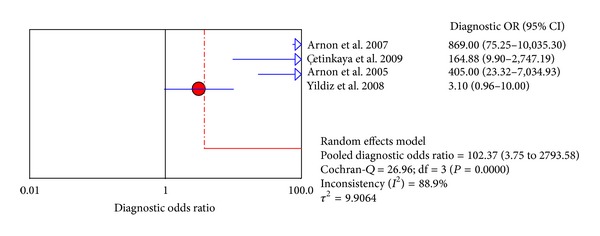
Forest plot for diagnostic OR of the SAA test to diagnose neonatal sepsis at 8–96 h after the first suspicion of sepsis.

Four studies from included papers evaluated the use of the SAA test at 8–96 h after the first suspicion of sepsis. Accordingly, Figure 6 shows the pooled sensitivity and specificity, and the diagnostic OR and SROC curve were shown in Figures 7 and 5, respectively.

### 3.3. Comparison of the Diagnostic Accuracy of Markers for NS

CRP and PCT have been proved to be useful biomarkers for the diagnosis of neonatal sepsis [15–17]. To show the value of diagnosis of the SAA test for NS, we compared SAA with CRP and PCT. Six trials evaluated the diagnosis of both SAA [21–24] and CRP [29, 30]. Compared with 0.67 (95% CI 0.62–0.73) for the CRP test, the pooled sensitivity for the SAA test was better (0.78 (95% CI 0.73–0.83)). 

Pooled specificity for the SAA test was slightly lower than for the CRP test, which was 0.89 (95% CI 0.84–0.92) versus 0.92 (95% CI 0.89–0.95). Their difference was not statistically significant (*P* < 0.05). However, the pooled diagnostic OR for the SAA test was smaller than that for the CRP test: 54.95 (95% CI 6.25–483.10) versus 77.16 (95% CI 9.79–248.21). Although this difference was statistically significant (*P* > 0.05), the Q* value for the SAA test was almost the same as that for the CRP test (0.89 VS 0.84), when the SROC curves for SAA and CRP tests were plotted, respectively. Meanwhile, the AUC for the detection of neonatal sepsis in the SAA test is larger than that in the CRP test (0.95 VS 0.91), which means that the SAA test is slightly better than CRP test for the diagnosis of NS by judging from the whole accuracy.

Two studies from included papers were selected to evaluate the use of the SAA and PCT for NS [23, 31]. The pooled sensitivity for the SAA test is slightly lower than for the PCT (0.78 (95% CI 0.73–0.82) versus 0.89 (95% CI 0.78–0.96)), but value of their specificity are similar to each other (0.87 versus 0.87). The pooled diagnosis OR for the SAA test was also almost similar to that for the PCT test (59.74 (95% CI 6.08–586.65) versus 60.17 (95% CI 8.09–447.31)). Since current trials are not sufficient to evaluate the use of SAA and PCT, the SROC curve for the PCT test cannot be plotted, and the Q* value and the AUC failed to be calculated. 

### 3.4. Analysis of Heterogeneity

In a meta-analysis of diagnostic test, heterogeneity is an important issue to understand the possible factors that influence accuracy diagnosis and estimate the appropriateness of statistical pooling of results from various studies. Variations are brought by several factors, such as the cutoff, assay method, and the age of patients.

In fact, heterogeneity may not be entirely avoided in meta-analysis, so it is necessary for us to explore the reason and extent of heterogeneity. Generally, one of the most important sources of heterogeneity is the threshold effect in a diagnostic study. So, we firstly explored the threshold effect, which was evaluated with the Spearman correlation coefficient with Moses' model weighted by inverse variance. We found that there was no statistically significant difference (Spearman's correlation coefficient = 0.133, *P*-value =0.731 > 0.5). The source of heterogeneity was explored by meta-regression analysis. The results show that cutoff (≥10 mg/L) is the largest factor (RDOR (relative diagnostic odds ratio) = 14.47, *P*-value = 0.0963), while the effects of the age (RDOR = 0.35, *P*-value =0.591), assay method (RDOR = 0.08, *P*-value =0.2405), region (RDOR = 0.5, *P*-value =0.4997), and QUADAS (RDOR = 1.54, *P*-value =0.8076) are small. The sensitivity analysis was also performed to identify further analysis of these sources. Homogeneity (*I* ^2^ = 0%) was showed among studies when we removed studies of Yildiz et al., 2008 [23], and Edgar, 2010 [24], including relatively small cutoff. If we only collect those studies in the diagnosis of late-onset neonates sepsis (postnatal age > 72 h), heterogeneity (*I* ^2^ = 89.9%) was found. When the study of Yildiz et al., 2008 [23] was removed, the remaining studies also showed homogeneity (*I* ^2^ = 0%). As the amount of the included studies is too small, we cannot do further analysis of other factors, such as subgroup analysis. We except that our results will become more convincing if more and more studies about the SAA test of the diagnosis of neonatal sepsis are published in the future.

### 3.5. Publication Bias

**Figure 8:**
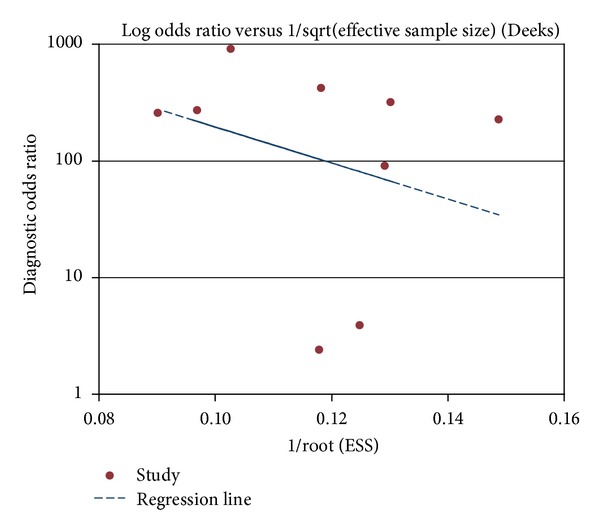
Test for the assessment of potential publication bias in the SAA test for the diagnosis of neonatal sepsis.

The Deeks test [32] was performed to detect publication bias by using the Stata 11 software. As shown in Figure 8, this was not statistically significant for the studies of the SAA test in the diagnosis of neonatal sepsis (*P* = 0.406 > 0.05). So, the result indicates no potential for publication bias. However, our included studies are so few that this result may be biased.

## 4. Discussion

Neonatal sepsis is the most common cause of neonatal deaths with high mortality; thus, its identification is vital to improve bad results. Clinical signs are subtle and nonspecific, and laboratory tests including blood culture are not always reliable [33]. So far, many markers for NS have been suggested, such as CRP, PCT, IL-6, and TNF-*α*. However, a single biomarker is not sufficiently reliable for identification of NS at present. More researchers focus on the combination of different biomarkers in different clinical settings and hope to achieve clearer conclusions [34, 35]. Therefore, it is necessary for us to clearly study important markers for future research of NS. Here, we demonstrated that the SAA test shows appropriate accuracy for the diagnosis of NS. 

An ideal biomarker should have high sensitivity and specificity, that is, have a high diagnostic accuracy [36]. In this meta-analysis, pooled sensitivity of the SAA test was 0.84 for the diagnosis of NS, the pooled specificity was 0.89, and Q* value was 0.91 at the first suspicion of sepsis; then, 8–96 h after the first suspicion of sepsis, a pooled sensitivity of the SAA test was 0.78 for the diagnosis of NS, the pooled specificity was 0.84, and Q* value was 0.91. These results show that the SAA test is a valuable biomarker and also has a long diagnostic cycle for the diagnosis of NS. 

Certainly, the limitations of our work should be considered. This meta-analysis only contained nine studies, though we tried our best to retrieve appropriate papers. In addition, we divided the sample into onset and 8–96 h after onset entirely based on characteristics of the sample, which accords with clinical facts, while the sample was divided into early-onset and late-onset neonatal sepsis generally. Different cut-off values, ranging from 1 to 75.17 mg/L, exist in the included studies. Publication bias might be generally difficult to be avoided in the meta-analysis, so we tried to include more studies and even adverse studies about the accuracy of the diagnosis of SAA to reduce the bias. To explore the reasons for heterogeneity, we considered the diagnostic cut-off point, which may partially explain this heterogeneity because of differences between studies. Therefore, we suspected that heterogeneity will be reduced by considering more studies for the SAA test, which might reduce the effect of different sampling, clinical settings, and other heterogeneity-caused factors.

CRP, a traditional useful marker, has been applied in clinic [37]. It is triggered by cytokines IL-6, TNF-*α*, and so forth and is a late marker of neonatal sepsis which increases evidently at 24 h after sepsis onset [22, 38]. Its sensitivity was 30%–97%, and its specificity ranged from 75% to 100% [39]. In included studies of the meta-analysis, the sensitivity of SAA varied from 26% to 100%, and specificity varied from 44% to 100%. To be truth, it is difficult to evaluate which marker is good or not. Lannergård et al. [40] also thought that there are positive correlations between SAA and CRP in infectious diseases. However, SAA rises earlier and more sharply than CRP, especially during the first 24 h after sepsis onset [41, 42], and it has showed a moderate accuracy at 8–96 h after the first suspicion of sepsis in our meta-analysis, which means that SAA is useful in the diagnosis of NS. 

In addition, PCT is also a useful marker which shows better accuracy than CRP for the diagnotic of NS in some aspects [16]. In our meta-analysis, the pooled diagnotic OR for the SAA test was also almost similar to that for the PCT test (59.74 versus 60.17). So, we believe that SAA is also a useful marker for the diagnosis of NS like CRP, PCT. Certainly, SAA cannot be replaced easily because it has a prognostic value as early as eight hours after the onset and before clinical signs. For example, des-arginine variants of SAA were identified as the most promising biomarkers, which can make neonatologists withhold treatment in 45% of nonsepsis neonates [43]. Moreover, SAA can be combined with sICAM-1, CRP, and sE-selectin to improve the diagnosis results, and it can be also added to PCT and CRP, which may increase the rate of sepsis diagnosis by about 10% [24, 29]. We believe that its usefulness will be showed if we evaluate it in combination with other markers or perform a clearer research on it alone in future studies.

In summary, SAA showed moderate accuracy and a longer diagnostic cycle in the diagnosis of neonatal sepsis. Furthermore, the SAA test has showed better accuracy than the CRP test for the diagnosis of neonatal sepsis in the first suspicion of sepsis. It not only has higher accuracy at the first suspicion of sepsis, but also keeps this usefulness at 8–96 after the first suspicion of sepsis. Accordingly, we believe that the combination of SAA with CRP and PCT will improve the diagnosis. To further analyze the diagnostic accuracy of the SAA test for the diagnosis of NS and correlation with other biomarkers in depth, follow-up clinical validation is needed.

## Figures and Tables

**Table 1:** Characteristics of studies included in the meta-analysis of the diagnosis of neonatal sepsis using a SAA test.

Study and year	Study population	Patients (*n*)	Region	Assay method	Time of SAA test: cutoff (mg/L)
Enguix et al. 2001 [31]	Cases: NICU neonates with sepsis	46	Spain	AMLN	Onset: 41.3
	Control: neonates without sepsis				
Arnon et al. 2007 [21]	Cases: full-term neonates with sepsis Control: neonates without sepsis	104	Israel	ALPIA	Onset: 8 24 h after the onset: 10
Çetinkaya et al. 2009 [29]	Cases: NICU neonates with probable sepsis Control: neonates without sepsis	163	Turkey	INMM	Onset: 68 48 h after the onset: 68
Yildiz et al. 2008 [23]	Cases: NICU newborns with suspected sepsis control: noninfected newborns	72	Turkey	ELISA	Onset: 5.5 96 h after the onset: 5.7
Arnon et al. 2005 [22]	Cases: neonates with proven or clinical sepsis Control: noninfected newborns	116	Israel	ELISA	Onset: 10 8 h after the onset: 10
Arnon et al. 2002 [44]	Cases: preterm infants with sepsis or suspected sepsis Control: healthy preterm infants	94	Israel	ELISA	Onset: 10
Edgar et al. 2010 [24]	Cases: term/preterm neonates with infection Control: term/preterm neonates with infection	68	England	ELISA	Onset: 1
Mostafa et al. 2011 [45]	Cases: infants with sepsis or suspected sepsis Control: healthy neonates	100	Egypt	ELISA	Onset: >10
Mohsen et al. 2012 [30]	Cases: term/preterm neonate with sepsis Control: healthy neonates	60	Egypt	ELISA	Onset: cases, 40.16 ± 35.17 control, 6.45 ± 2.42

AMLN: automatic laser nephelometry; ALPIA: automated latex photometric immunoassay; INMM: immunonephelometric method; ELISA: enzyme-linked immunoassay; HSAIA: highly sensitive automated immunoassays, and NICU: neonatal intensive care unit.

**Table 2:** True positive, Fp, Fn, Tn, Se, Sp, time, and QUADAS of included studies for the diagnosis of NS.

Study and year	Tp	Fp	Fn	Tn	Se	Sp	Time	QUADAS
Enguix et al. 2001 [31]	19	2	1	24	0.95	0.92	Onset	9
Arnon et al. 2007 [21]	22	4	1	77	0.96	0.95	Onset	7
	22	2	1	79	0.96	0.98	24 h after the onset	
Çetinkaya et al. 2009 [29]	94	0	29	40	0.76	1	Onset	6
	80	0	41	42	0.66	1	48 h after the onset	
Yildiz et al. 2008 [23]	27	20	9	16	0.75	0.44	Onset	5
	31	24	5	12	0.86	0.33	96 h after the onset	
Arnon et al. 2005 [22]	40	5	2	68	0.95	0.93	Onset	8
	37	12	0	67	1	0.85	8 h after the onset	
Arnon et al. 2002 [44]	42	4	0	48	1	0.92	Onset	6
Edgar et al. 2010 [24]	6	3	20	39	0.23	0.93	Onset	8
Mostafa et al. 2011 [45]	80	2	2	16	0.98	0.89	Onset	6
Mohsen et al. 2012 [30]	26	2	4	28	0.87	0.93	Onset	6

Tp: true positive; Fp: false positive; Fn: false negative; Tn: true negative; Se: sensitivity; and Sp: specificity.
